# Experiences of a tertiary center with use of extracorporeal membrane oxygenation support in patients with cardiogenic shock after cardiac surgery

**DOI:** 10.1186/cc13356

**Published:** 2014-03-17

**Authors:** L Galas, V Guimaraes, M Sundin, L Caneo, M Jatene, F Jatene, L Hajjar, J Almeida, F Galas

**Affiliations:** 1 Instituto do Cancer do Estado de São Paulo, Brazil; 2 Heart Institute, São Paulo, Brazil

## Introduction

Profound myocardial depression can occur after cardiac surgery. Use of ventricular assist support through venoarterial extracorporeal membrane oxygenation (ECMO) has been positively reported. This study will focus on the outcomes of patients who, upon suffering hemodynamic failure after cardiac surgery, were supported by the use of ECMO during their stay in the surgical ICU of Incor FMUSP.

## Methods

This was a retrospective, single-center and observational study. The records of 48 patients who underwent cardiac surgery and, subsequently, needed percutaneous or surgical implantation of ventricular assist devices were evaluated. The evaluation considered the following criteria: basal characteristics, indications for ventricular assistance, duration, length of ICU and hospital stay, and hospital mortality, through data collection forms.

## Results

Of the 48 patients included on the study, 26 (54%) were males, and 31 (64%) were younger than 18 years old. These patients developed cardiogenic shock during 72 hours after cardiac surgery. In all cases, ECMO was inserted after cardiac surgery. Of all patients, 32 (66%) were central ECMO, inserted in the operative room, and 16 were percutaneous, inserted in the ICU. The median duration of ventricular assistance was 6 days (IQR 0 to 41), the length of ICU stay was 16 days (IQR 1 to 111), and hospital stay was 29 days (IQR 1 to 198). Twenty patients survived (41%) and were discharged from our hospital.

## Conclusion

The use of mechanical circulatory assists devices is an efficient tool to manage seriously ill patients after cardiac surgery. This tool should be considered early in the diagnosis of cardiogenic shock after cardiac surgery.

